# Experimental inoculation trial to determine the effects of temperature and humidity on White-nose Syndrome in hibernating bats

**DOI:** 10.1038/s41598-022-04965-x

**Published:** 2022-01-19

**Authors:** Winifred F. Frick, Emily Johnson, Tina L. Cheng, Julia S. Lankton, Robin Warne, Jason Dallas, Katy L. Parise, Jeffrey T. Foster, Justin G. Boyles, Liam P. McGuire

**Affiliations:** 1 Bat Conservation International, Austin, TX 78746 USA; 2 Department of Ecology and Evolutionary Biology, University of California, Santa Cruz, CA 95060 USA; 3 Department of Biological Sciences, Texas Tech University, Lubbock, TX 79401 USA; 4 U.S. Geological Survey - National Wildlife Health Center, Madison, WI 53711 USA; 5 Cooperative Wildlife Research Laboratory and School of Biological Sciences, Southern Illinois University, Carbondale, IL 62901 USA; 6 Pathogen and Microbiome Institute, Northern Arizona University, Flagstaff, AZ 86011 USA; 7 Department of Biology, University of Waterloo, Waterloo, N2L 3G1 Canada

**Keywords:** Ecology, Behavioural ecology, Conservation biology, Ecological epidemiology

## Abstract

Disease results from interactions among the host, pathogen, and environment. Inoculation trials can quantify interactions among these players and explain aspects of disease ecology to inform management in variable and dynamic natural environments. White-nose Syndrome, a disease caused by the fungal pathogen, *Pseudogymnoascus destructans* (*Pd*), has caused severe population declines of several bat species in North America. We conducted the first experimental infection trial on the tri-colored bat, *Perimyotis subflavus*, to test the effect of temperature and humidity on disease severity. We also tested the effects of temperature and humidity on fungal growth and persistence on substrates. Unexpectedly, only 37% (35/95) of bats experimentally inoculated with *Pd* at the start of the experiment showed any infection response or disease symptoms after 83 days of captive hibernation. There was no evidence that temperature or humidity influenced infection response. Temperature had a strong effect on fungal growth on media plates, but the influence of humidity was more variable and uncertain. Designing laboratory studies to maximize research outcomes would be beneficial given the high costs of such efforts and potential for unexpected outcomes. Understanding the influence of microclimates on host–pathogen interactions remains an important consideration for managing wildlife diseases, particularly in variable environments.

## Introduction

Infectious disease can threaten wildlife populations by causing rapid population declines, particularly when pathogens first emerge or are introduced into novel areas with naïve hosts^1–3^. Research to determine whether management actions are effective at reducing disease effects can be challenging to conduct, especially in dynamic and complex ecological systems^4^. Controlled experiments are often used to determine disease susceptibility of hosts or quantify aspects of host–pathogen-environment interactions^5^. Experimental inoculation trials in laboratory settings provide the advantage of controlling and randomly assigning specific conditions of interest to enable causal inference and determine disease susceptibility. Ideally, these inferences can be used to explain key aspects of disease ecology and inform management actions taking place in variable and dynamic natural environments^4^.

White-nose Syndrome (WNS) is an infectious disease of hibernating bats caused by a fungal pathogen (*Pseudogymnoascus destructans*; *Pd*)^6–9^ that was first detected in eastern North America in 2006^10^ and has caused rapid and extreme population declines of several species of hibernating bats^11–14^. Over the past 15 years, extensive research has revealed much about the disease ecology of WNS, including the interactions between the pathogen, environments where bats hibernate, and the physiology of hibernating bats (see^12^ for recent comprehensive review). Research efforts have also focused on testing potential management actions aimed to reduce transmission or spread, lower disease severity, or improve survival^15–18^. One of the challenging aspects of managing WNS is that the pathogen persists in the environment where bats hibernate, exposing bats to infection at the start of each hibernation season^19,20^. Understanding the specific conditions that affect disease severity and population declines where the pathogen invades and establishes^12,19,21,22^ remains important for informing management response^4,11^.

Multiple studies on hibernating bats indicate that impacts from WNS may be influenced by both temperature and humidity conditions where bats hibernate^22–25^, most likely due to the complex relationships between microclimates, hibernation physiology, and *Pd* fungal growth^12,24,26,27^. In areas where *Pd* has established in North America, fungal loads are positively correlated with roosting temperatures of hibernating species, such that species that tend to roost at relatively warmer temperatures tend to have higher loads and in turn higher mortality from WNS^24^. In vitro experiments show that *Pd* growth is temperature dependent with highest growth rates between 12 and16 °C^26^. Humidity can also influence *Pd* growth^28^, but has been harder to study in situ due to the difficulty of reliably measuring humidity in natural hibernacula^29^.

*Perimyotis subflavus* has experienced an overall 93% population decline across the range of where WNS has established and the species is currently being evaluated for regulatory protection under the Endangered Species Act^11^. Although the effects of WNS on this species have been well-documented in numerous field studies^11,12,14,21,23^, no experimental studies had yet been conducted to examine the species’ hibernation physiology and disease susceptibility. Most experimental infection trials on WNS have been conducted with little brown bats (*Myotis lucifugus*)^8,9,15,30–33^ and there is a recognized need to study the interactions among host, pathogen, and environmental conditions in other bat species whose populations are declining from WNS^12^. In natural habitats, *P. subflavus* hibernate across a range of environmental conditions, including at temperatures associated with high *Pd* growth rates and high disease mortality^22,24,26,29,34^.

We conducted the first experimental infection trial on *P. subflavus* to test the effect of temperature and humidity conditions on infection and disease severity of WNS. We asked three questions: (1) What is the effect of temperature and humidity on WNS disease severity of *P. subflavus*? (2) Does pre-hibernation condition of bats influence disease outcome? and (3) What is the effect of temperature and humidity on growth of *Pd* on environmental substrates? We predicted that disease severity would be higher at warmer and more humid microclimate conditions based on evidence from field studies that indicate these conditions are associated with higher fungal loads and more severe declines in winter colony size after *Pd* arrival (reviewed in^12^). We designed the experiment to determine behavioral selection of specific temperature and humidity conditions. Most natural hibernacula have a range of temperature and humidity^29^ and behavioral selection by bats of beneficial microclimates would be necessary if management strategies of providing thermal refugia are to succeed^22,35^. We hypothesized that pre-hibernation body condition, specifically fat mass and stress indicator (measured by fur cortisol) would be related to disease outcomes. We also addressed how microclimate conditions may affect the environmental reservoir of *Pd* given its important role in transmission dynamics^19^. Using the same environmental chambers and microclimate conditions, we conducted a separate experiment testing the effects of temperature and humidity gradients on fungal growth and persistence on artificial and natural substrates.

## Methods

All methods in this study were approved by the Institutional Animal Care and Use Committee at Texas Tech University (protocol 18032-12). All procedures were performed in accordance with relevant guidelines in the manuscript and the ARRIVE (Animal Research: Reporting of In Vivo Experiments) guidelines (https://arriveguidelines.org/).

### Experimental design for testing effects of temperature and humidity on Pd infection severity on *Perimyotis subflavus*

**Figure 1:**
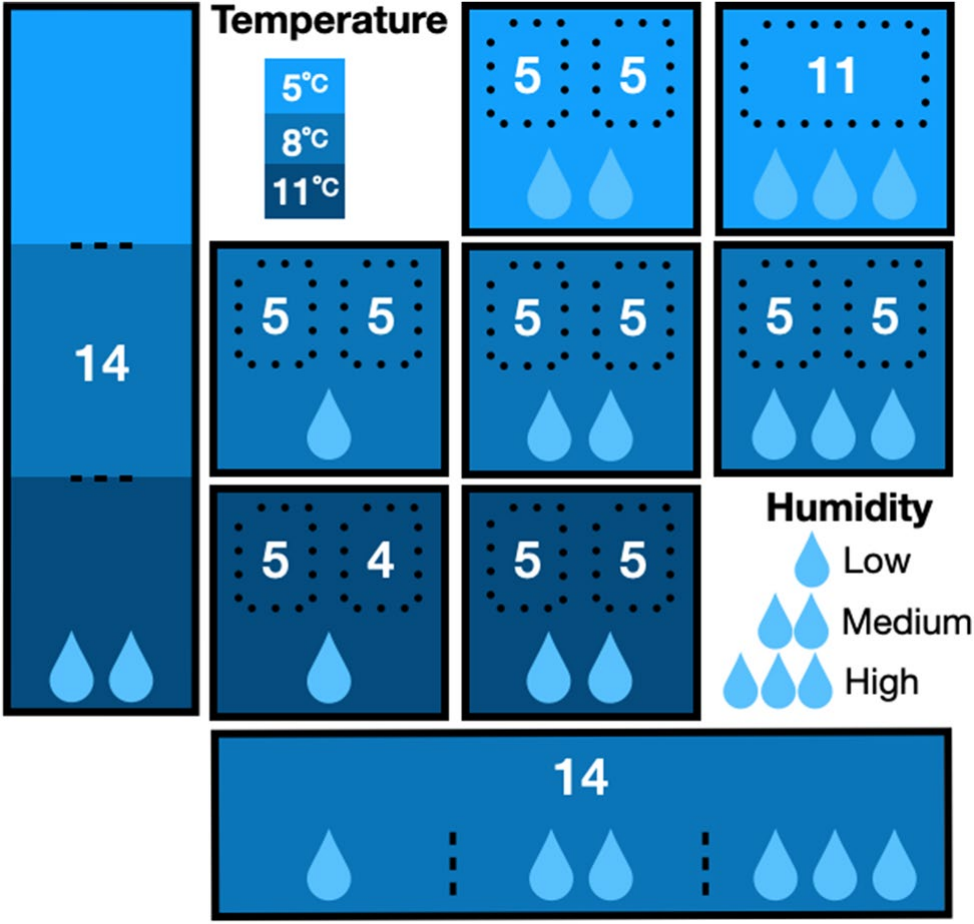
Schematic of the experimental design and sample sizes with 7 environmental chambers with fixed temperature and humidity conditions and two sets of connected chambers allowing bats to behaviorally select temperature (left) or humidity conditions (bottom) for the infection trial on tri-colored bats (*Perimyotis subflavus*). Water loss conditions were based on water vapor pressure deficit (VPD) levels set to 0.05 kPA to produce low potential evaporative water loss (pEWL) for high humidity, 0.10 kPa for medium pEWL and humidity, or 0.15 kPA for high pEWL and low humidity. Numbers are sample sizes of bats assigned to separate cages within each chamber. Bats in the low temperature and high humidity chamber were combined into a single cage after a camera failed at the start of the experiment (top right).

We randomly assigned bats to seven environmental chambers (Caron, Model 7000-33-1, Marietta, Ohio, USA) in a blocked experimental design, controlling temperature and humidity in each chamber (Fig. 1). In each environmental chamber, we divided bats into two cages (23 × 38 × 50 cm) constructed from mesh fabric (Part FMLF, Seattle Fabrics, Inc., Seattle, Washington, USA), PVC pipe, and plastic sheeting. We stratified random assignment to ensure even distribution of initial body mass and sex across microclimate treatments. In addition to the seven treatments with fixed temperature and humidity conditions, we had two treatments that allowed bats to freely move among temperature or humidity conditions (Fig. 1). One group of bats (n = 14) was free to move among three chambers with a common temperature (8 °C) but different humidity (water vapor pressure deficit (VPD) = 0.05 kPa, 0.10 kPa, or 0.15 kPa, corresponding to 95, 90, and 85% relative humidity (RH))^36^. A second group of bats (n = 14) was free to move among three chambers with a common VPD condition (0.10 kPa, medium humidity) but different temperatures (5, 8, or 11 °C) (Fig. 1). Because our research questions were focused on comparing the effect of temperature and humidity conditions on disease severity, we did not include sham-inoculated control animals in the experiment. We made this decision to reduce the total number of animals used in the experiment and to maximize replication to test the effects of temperature and humidity on disease.

We inoculated each bat by spreading 20 µL of *Pd* solution (5 × 10^5^ conidia µL^−1^) evenly across both wings, following established protocols^8,9,32,37^; treatments were conducted blind without knowledge of which bat was being assigned to what group and bats were inoculated in no particular order to reduce the confounding influence on the order of treatment. We used a *Pd* strain collected by Karen J. Vanderwolf at Trent University from naturally infected *Myotis lucifugus*. We cultured *Pd* on Sabouraud Dextrose Agar with chloramphenicol and gentamicin (SabDex) (Part L96359, Fisher Scientific, Houston, Texas, USA) and incubated subcultured plates at 10 °C for 60 days to allow the formation of conidia. We then harvested conidia by flooding plates with phosphate buffered saline solution containing 0.5% Tween20 (PBST). Conidia were resuspended in PBST, enumerated, and diluted to the inoculum concentration^8^.

### Microclimate treatment conditions

We used three temperatures 5, 8, or 11 °C to represent a range of roosting temperatures of *P. subflavus* in natural hibernacula^24,29^. We set humidity in environmental chambers to achieve specific levels of water vapor pressure deficit (VPD) between the surface of the bat and the environment because relative humidity varies by temperature^36^. Higher VPD corresponds to drier air resulting in higher potential evaporative water loss (pEWL). We used three levels of VPD: 0.05, 0.10, or 0.15 kPa corresponding to low pEWL (high humidity), medium pEWL (medium humidity), and high pEWL (low humidity) levels (Fig. 1). We verified the ambient temperature and relative humidity in each chamber at 10-min intervals (Hobo Model U23-001, Onset Computer Corporation, Bourne, Massachussetts, USA). For bats in the connected chambers that could behaviorally select their temperature and humidity conditions, we quantified the number of days bats spent in each condition^38^.

### Animal handling and data collection

We used 98 (42 females, 56 males) tricolored bats collected on 10 December 2018 from culverts in Mississippi and transported directly to Texas Tech University^39^. We took morphometric measurements (body mass ± 0.1 g, forearm length ± 0.1 mm) and used quantitative magnetic resonance (QMR; Echo-MRI-B, Echo Medical Systems, Houston, Texas, USA) to determine pre-hibernation fat at the start of the experiment^39,40^. As an indicator of pre-hibernation stress, we collected a fur sample from the dorsal intrascapular region to quantify fur cortisol concentration with a commercial ELISA kit, following the manufacturer’s protocol (Arbor Assays, Michigan, USA) (see Supplemental Methods). Fur is moulted once per year in the late summer period^41^ and therefore fur cortisol reflects the level of circulating cortisol during the period of fur growth prior to hibernation. We attached a uniquely marked, modified datalogger^42^ (DS1925L iButton, Maxim Integrated, San Jose, California, USA) to the back of each bat using ostomy cement to record skin temperature^39^. Prior to inoculation, we swabbed bats with a sterile polyester swab (Fisherbrand synthetic tipped applicators 23-400-116) five times on forearm and five times on muzzle to determine if any bats were naturally infected with *Pd* at time of collection. Swabs were stored in RNAlater at  − 20 °C until testing using quantitative polymerase chain reaction (qPCR) at Northern Arizona University^43^.

During the experiment, we provided ad libitum drinking water in each cage but did not provide food. We secured a motion-activated infrared camera (Model HT5940T, Speco Technologies, New York, New York, USA) above each cage to monitor bats throughout the experiment. Because one camera failed at the start of the experiment, we combined bats in that treatment chamber into a single cage (Fig. 1) and replicated this disturbance among all chambers. We monitored bats without disturbance by reviewing video recordings daily. Three bats died of unknown cause before the end of the experiment and were removed from analyses.

After 83 days of hibernation, we terminated the experiment and bats were removed from cages and processed to determine body condition using QMR^39^. We took respirometry measurements on a subset of animals^38^, and swabbed for *Pd* as described above. We photographed the left ventral wing using ultraviolet (UV) transillumination (368-nm wavelength and 2-s exposure) to detect and measure florescence associated with *Pd* infection^37,44^. For histology, we removed the wing section from the fifth digit and the body and rolled wing tissue around dental wax dowels and 10% neutral buffered formalin. We collected a 90–110 µL blood sample in lithium-heparin-treated capillary tubes for immediate analysis of blood chemistry with a handheld analyzer (i-STAT1 Vet Scan, Abaxis, Union City, California, USA). Using an EC8+ cartridge, we measured sodium, potassium, chloride, anion gap, glucose, BUN (urea nitrogen), hematocrit, hemoglobin, pH, pCO2, TCO2, HCO3, and base excess (Table S1). We quantified arousals from torpor as reported by McGuire et al.^39^. All bats were handled and euthanized under Animal Care and Use Committee permit 18032-12 at Texas Tech University.

### Infection and disease metrics

We used several metrics to determine pathogen and disease presence and severity^37^: presence and amount of the pathogen, *Pd,* on a bat were determined by qPCR^43^, and presence of the disease, WNS, was determined via detection of orange-yellow florescence under UV light characteristic of *Pd* infection^44^ and histological presence of characteristic lesions and pustules with fungal hyphae^45,46^. Three types of cutaneous infection were described histologically, including characteristic cupping erosions with fungal hyphae, neutrophilic pustules with fungal hyphae, and fungal hyphae in the stratum corneum with dermal necrosis. Any bats with any of these three conditions noted were scored as WNS positive by histology. Presence and quantity of DNA of *Pd* was tested by qPCR at Northern Arizona University. All samples were run in duplicate and considered positive if at least one run was positive below a cycle threshold (Ct) of 40 and quantified using a quantification curve from serial dilutions (nanograms of *Pd* using the equation load = 10^((22.049-Ct value)/3.34789)^, r^2^ = 0.986)^47^. Load values were averaged across multiple runs and then converted to attograms by multiplying loads in nanograms by 10^9^.

### Statistical analyses

**Figure 2:**
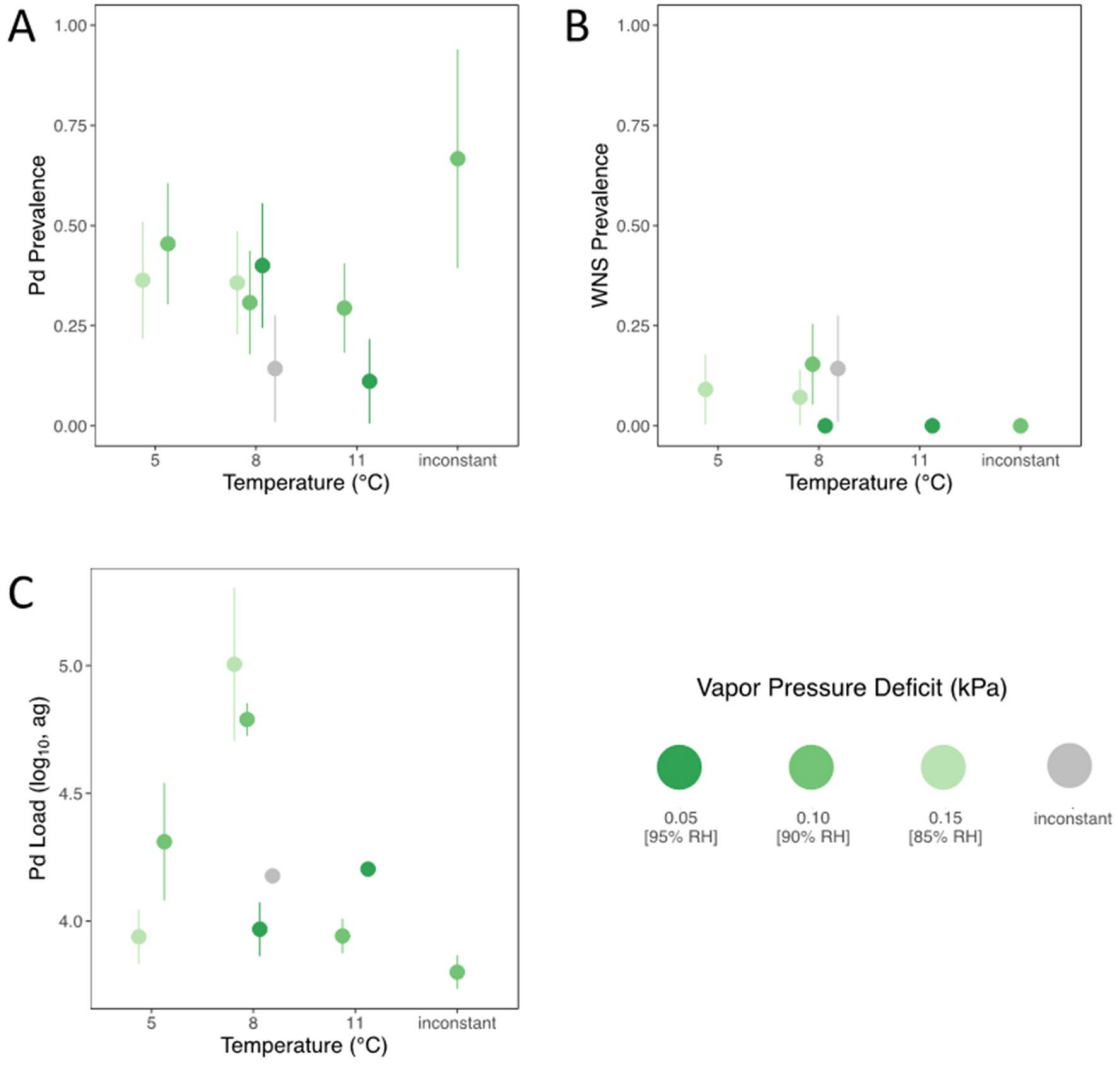
Signs of *Pseudogymnoascus destructans* (*Pd*) infection or white-nose syndrome (WNS) disease for tri-colored bats (*Perimyotis subflavus*) exposed to different temperature and humidity regimes. (**A**) Fraction of bats with *Pd* detected by qPCR; (**B**) Fraction of bats with signs of WNS disease by histology, and (**C**) Mean quantity of *Pd* on bats at the end of the experiment. There was no statistical support for differences between temperature or humidity treatments for any response metrics. Points are estimated means and vertical lines show binomial standard error for prevalence and standard errors for *Pd* load.

We used three different response variables (*Pd* prevalence, *Pd* loads, and WNS prevalence by histology) to determine whether infection status varied by microclimate treatment conditions. Low sample sizes of positive infection status by UV detection (n = 4) precluded use in statistical analyses (Table 1). We used generalized linear models with binomial distribution for analyses of *Pd* prevalence and WNS prevalence and a linear mixed effects model with Gaussian errors for *Pd* loads. Although the experiment was designed with replication at the cage level to account for cage effects, we were unable to include cage as a random effect because of the low numbers of bats that had signs of *Pd* or WNS infection. We analyzed whether infection status (i.e., *Pd* prevalence, *Pd* load, or WNS prevalence) varied by sex and cortisol separately from an a priori candidate model set (Table 2) to cope efficiently with small sample sizes. We first asked whether infection response varied by sex to determine if bats could be pooled in subsequent analyses. We analyzed separately whether infection response varied by pre-hibernation cortisol at the start of the experiment on the subset of animals for which we had cortisol measurements (n = 83). We then used an information-theoretic approach comparing a candidate set of models with Akaike Information Criterion (AIC)^48^ using initial fat mass as an individual covariate and temperature and humidity treatment conditions as categorical treatment groups to assess the effect of microclimate on infection response (Table 2). Bats behaviorally selecting their temperature and humidity conditions were assigned to a temperature or humidity treatment level if a bat spent > 89% of captive days at that condition or was otherwise placed in an ‘inconstant condition’ treatment group. For WNS prevalence, we used the bias reduction method implemented in package brglm^49^ to deal with complete separation present in the data (in some treatments all bats were scored as negative for WNS) (Table 1; Fig. 2).

**Table 1:** Signs of *Pd* infection or WNS disease for tri-colored bats (*Perimyotis subflavus*) exposed to different temperature and humidity regimes.

Temperature	Humidity	*Pd* prevalence qPCR	WNS prevalence histology	WNS prevalence UV	Overall *Pd*/WNS prevalence	*Pd* load
Low (5 °C)	Medium (0.1 kPa)	45% (5/11)	18% (2/11)	0% (0/11)	55% (6/11)	4.31 (0.34)
	High (0.05 kPa)	36% (4/11)	9% (1/11)	0% (0/11)	45% (5/11)	3.94 (0.17)
Medium (8 °C)	Low (0.15 kPa)	40% (4/10)	0% (0/10)	0% (0/10)	40% (4/10)	3.97 (0.16)
	Medium (0.1 kPa)	31% (4/13)	15% (2/13)	15% (2/13)	31% (4/13)	4.79 (0.11)
	High (0.05 kPa)	36% (5/14)	7% (1/14)	7% (1/14)	36% (5/14)	5.01 (0.5)
	Inconstant (0.05–015 kPa)	14% (1/7)	14% (1/7)	0% (0/7)	14% (1/7)	4.18 (*)
High (11 °C)	Low (0.15 kPa)	11% (1/9)	0% (0/9)	0% (0/9)	11% (1/9)	4.2 (*)
	Medium (0.1 kPa)	29% (5/17)	18% (3/17)	6% (1/17)	41% (7/17)	3.94 (0.12)
Inconstant (5–11 °C)	Medium (0.1 kPa)	67% (2/3)	0% (0/3)	0% (0/3)	67% (2/3)	3.8 (0.08)
	Total	33% (31/95)	11% (10/95)	4% (4/95)	37% (35/95)	4.29 (0.12)

We report the proportion of bats with positive detections of *Pseudogymnoascus destructans* (*Pd*) detected by qPCR (*Pd* Prevalence by qPCR, the proportion of bats with signs of *Pd* infection from histological examination (WNS Prevalence histology) and by fluorescent cutaneous infection visible by ultraviolet light (WNS Prevalence UV). We report the mean *Pd* load in attograms with standard error in parentheses; asterisk (*) indicates a single estimate in some groups. For bats that could select a temperature or humidity during the experiment, individuals that spent > 89% of captive days in a chosen temperature or humidity were assigned to a treatment group, otherwise they were assigned to an inconstant group representing variable conditions.

**Table 2:** Model selection results for model comparisons of humidity and temperature and pre-hibernation fat mass on *Pd* prevalence, *Pd* load, and WNS prevalence.

Model formula	*Pd* prevalence			*Pd* load			WNS prevalence		
	df	AIC	ΔAIC	df	AIC	ΔAIC	df	AIC	ΔAIC
[NULL MODEL]	1	121.99	1.1	3	65.97	0	1	65.48	0
Fat	2	120.89	0	4	67.32	1.35	2	65.79	0.31
Humidity	4	126	5.1	6	69.65	3.68	4	66.68	1.2
Temp	4	124.72	3.83	6	68.7	2.72	4	70.38	4.89
Fat + humidity	5	124.64	3.75	7	71.05	5.08	5	67.18	1.7
Fat + temp	5	123	2.1	7	70.24	4.27	5	70.45	4.97
Humidity + temp	7	129.14	8.25	9	70.26	4.29	7	72.45	6.96

Because this was the first captive hibernation experiment with *P. subflavus*, we investigated the effects of temperature and humidity on the hibernation physiology of the species^38,39^ and how physiological markers (e.g., blood chemistry) may be associated with disease. To determine if physiological indicators were related to infection status at the end of the experiment, we compared total number of torpor arousal bouts during the experiment and 13 different blood chemistry metrics from blood samples taken at the end of the experiment and used t-test comparisons (at α = 0.05) for each metric between *Pd*/WNS positive and negative bats. We designated bats as *Pd*/WNS positive if a bat tested positive for either *Pd* or WNS by qPCR, UV, or histology. We used Program R version 3.6.2 to conduct all analyses.

### Experimental design for testing effects of temperature and humidity on Pd growth on substrates

We used five environmental chambers (CARON, Model 7000-33-1, Marietta, Ohio, USA) to test for the effects of temperature and humidity on fungal growth on natural and artificial substrates (Fig. S1). Our experimental design comprised a reduced temperature series and humidity gradient than what we used for the experiment on bats. In the humidity gradient, temperature was held constant at 8 °C, with 85%, 90%, and 95% RH representing our low, medium, and high humidity treatments, respectively. In the temperature series, vapor pressure deficit (VPD) was held constant across the low (5 °C), medium (8 °C), and high (11 °C) temperatures (VPD = nominally 0.01 kPa, range (0.105–0.107). The chamber set to 8 °C and 90% humidity (VPD = 0.107 kPa) was common to both series.

### Media plate inoculation and fungal growth measurement

We constructed modified plate lids to prevent contamination while allowing humidity to equilibrate across the plate lid. We drilled 14 equidistant holes (5.5 mm diameter) into each plate lid and hot glued a piece of circular filter paper to the top of the lid. Lids were then disinfected thoroughly with a hydrogen peroxide wipe before being placed in a disinfected, sealed storage container.

We prepared *Pd* inoculum as described above for the infection trial on bats. We inoculated 30 SabDex plates with 100 µL of inoculum at a concentration of 20 conidia µL^−1^ by serial dilution with a starting concentration of 2.0 × 10^4^ conidia µL^−1^ diluted four times by a factor of 10. We used sterile, individually wrapped 1-µL plastic inoculation loops to spread the inoculum evenly across the surface of the plates, added the modified plate lids, and immediately transferred plates into environmental chambers. We included six replicate plates in each of the five microclimate conditions.

**Figure 3:**
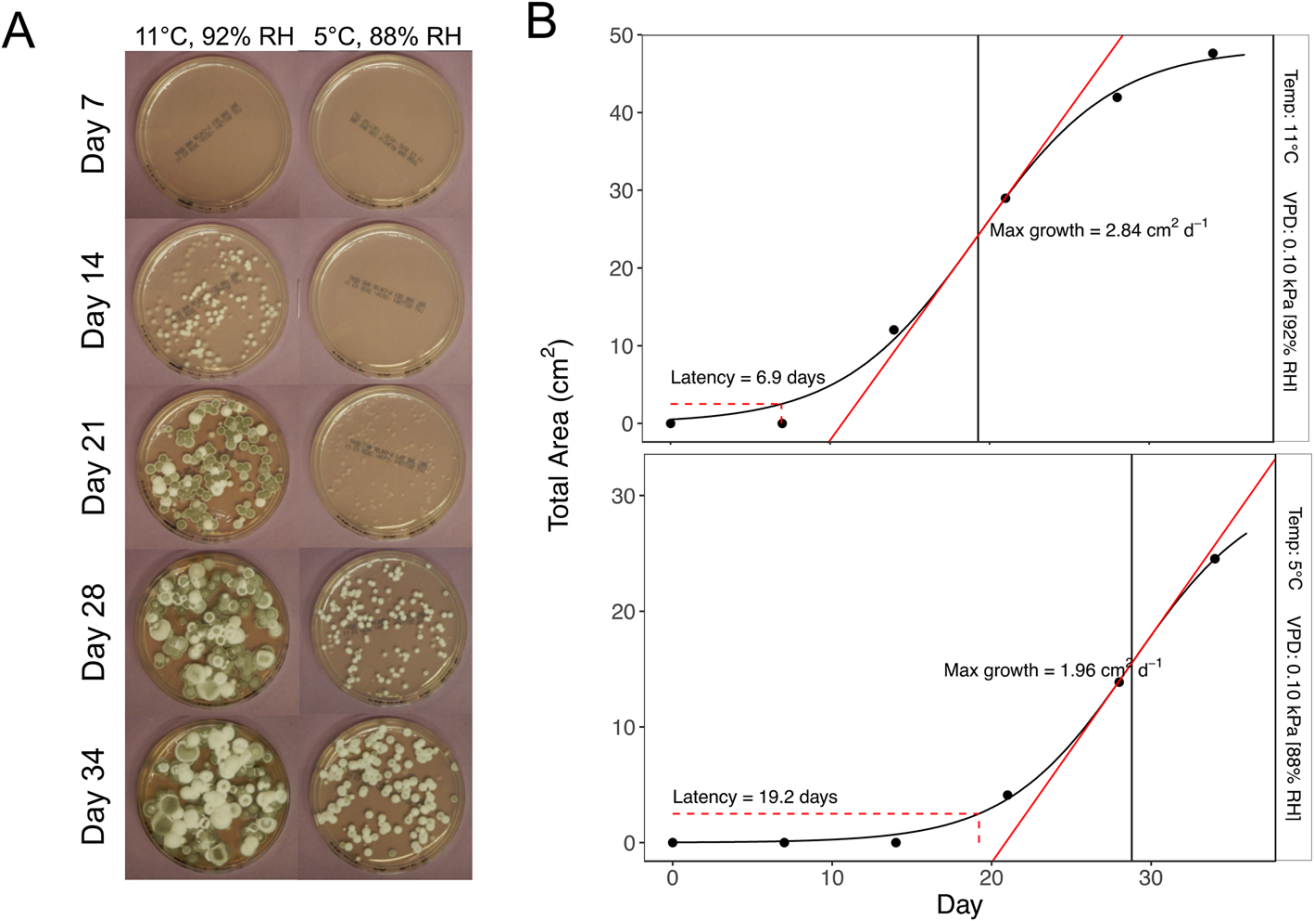
Examples demonstrate the process of measuring and estimating fungal growth of *Pseudogymnoascus destructans* (*Pd*) on media plates in temperature and humidity treatment conditions. (**A**) Examples of fungal growth on media plates measured at days 7, 14, 21, 28, and 34 from two of the treatment conditions (11 °C, 92% RH and 5 °C, 88% RH). (**B**) Examples of estimating maximum growth rate and latency variables from fungal growth measurements in panel A. We fit a sigmoidal curve to describe fungal growth (thick solid black line) to estimate the inflection point of the curve (vertical solid line). We calculated the slope (solid red line) at the inflection point of the curve to estimate maximum growth rate, and the days until total growth area reached 2.5 cm^2^ (dashed red lines) as an estimate of latency.

We took weekly digital photographs (Nikon, Model 26524, Tokyo, Japan) of each plate for the 5-week duration of the experiment (Fig. 3A). Our camera was mounted on a tripod to ensure consistent placement of plates relative to the camera. Each photo included a ruler, which was used to calibrate measurements made in ImageJ (Version 2.0.0-rc-69/1.52p, National Institutes of Health, Bethesda, Maryland, USA). One observer made all measurements for consistency. We used the freehand selection tool to trace the boundary of each fungal colony using a drawing tablet (Wacom, Model CTL-490, Kazo, Saitama, Japan). From these selections, we obtained the total surface area growth as the sum of all area selection (in cm^2^).

We modelled the growth of *Pd* on each plate as a sigmoidal curve (Fig. 3B), which we fit using the SSlogis and nls functions in Program R v. 3.6.3^50^. The model fitting function provides an estimate of the inflection point of the curve, and we calculated the slope at the inflection point to estimate the maximum growth rate. We also estimated the latency to rapid fungal growth on the plates by determining the date at which the total area of fungus on the plate reached 2.5 cm^2^ as an arbitrary threshold.

We also quantified growth of individual colonies. To avoid biasing growth rate estimates, we excluded colonies that intercepted another colony by choosing independent colonies at the final time point and tracking them backwards through time. If there were fewer than 10 independent colonies at the final time point, we added additional unimpeded colonies with each earlier time point until the total number of colonies reached 10. We modelled growth of individual colonies following the same procedure as for total area of growth on the plate, with an arbitrary threshold of 0.05 cm^2^ for latency calculations. We used linear mixed models to test for the effects of temperature and humidity on maximum growth rate or latency, including plate as a random factor to account for measuring multiple colonies per plate.

### Rock inoculation and fungal growth measurement

To evaluate fungal growth and persistence on a natural substrate, we inoculated pieces of sandstone flagstone. We etched a 4 × 6 sampling grid, composed of 5 × 5 cm squares, onto the surface of each sandstone rock (Texas Rock and Flagstone, Lubbock, Texas, USA), where each square served as a sampling unit (Fig. S2). Each row represented a time series for a single replicate, while each column was composed of replicates for the respective time point. Rocks were then autoclaved at 121 °C for 40 min and stored individually in a disinfected, sealed container until inoculation. At the time of inoculation, we evenly spread 200 µL of inoculum (2.5 × 10^4^ conidia µL^−1^) across each sampling square and immediately transferred the rock to an environmental chamber.

We measured fungal growth at days 0, 14, 28, and 56. We used a sterile cotton swab to collect fungal DNA from each sampling square. Swabs were moistened with RNAlater and rolled horizontally, vertically, and diagonally across the surface of the sampling square to ensure contact with the total surface area. One researcher collected all swabs to maximize consistency among swabs collected throughout the experiment. Swabs were placed in RNAlater and stored at − 20 °C until shipped to Northern Arizona University for qPCR analysis^43^. We quantified fungal loads for each swab sample from qPCR using the quantification curve provided above and normalized fungal loads to the value at day zero for each rock respectively. We then used linear models to test for effects of temperature and humidity on changes in fungal load (log transformed) over time.

To evaluate viability of *Pd*, we swabbed the entire inoculated surface of each rock at the end of the experiment and vortexed the swabs in RNAlater for one minute to release fungal DNA from the swab. We then applied 100 µL of RNAlater fungal solution from each rock to a respective SabDex media plate, using a sterile inoculation loop. After 2 weeks of incubation at 11 °C and 92% RH, we visually assessed plates for presence of fungal growth to determine viability of *Pd* collected from rocks at the end of the growth experiment.

## Results

### Experimental infection trial on *Perimyotis subflavus*

Only 37% (35/95) of the bats showed any infection response by the end of the experiment with 33% (31/95) testing positive for *Pd* by qPCR, 11% (10/95) scored as positive by histology, and only four bats showed signs of *Pd* infection by UV illumination (Table 1). Of 10 bats scored as positive by histology, three bats had cupping erosions containing fungal hyphae and seven bats had either neutrophilic pustules containing fungal hyphae, dermal necrosis associated with intra-epidermal fungal hyphae, or both.

Of the 14 bats that could behaviorally select temperature condition, seven bats spent > 89% of their captive days in the 11 °C chamber (4/7 spent 100% of captive days), one bat spent 100% of captive days in the 5 °C chamber, and three bats moved between chambers spending < 60% of captive days in any one temperature condition. Of the 14 bats that behaviorally selected humidity conditions, four bats spent 100% of captive days at the low pEWL/high humidity condition (0.05 kPA), three bats spent > 89% (2/3 spent 100%) of captive days at medium pEWL/humidity (0.10 kPA), and seven bats moved among chambers and were inconstant in selecting their humidity conditions. Detailed results on behavioral selection of microclimate conditions by bats in this study are provided in^38^.

We found no evidence for a difference in *Pd* infection response between males and females (likelihood ratio test (LRT): *Pd* prevalence: χ^2^ = 1.44, df = 1, P = 0.23; *Pd* load: χ^2^ = 0.01, df = 1, P = 0.92) nor any evidence of a relationship with pre-hibernation cortisol (LRT: *Pd* prevalence: χ^2^ = 0.49, df = 1, P = 0.48; *Pd* load: χ^2^ = 1.04, df = 1, P = 0.31). There was some support that WNS disease by histology differed between males and females (LRT: χ^2^ = 6.17, df = 1, P = 0.02) as 16% (n = 9/55) of males scored positive by histology compared to 2% (n = 1/44) of females. Because only one female scored positive by histology, bats of both sexes were pooled in subsequent analyses of WNS prevalence. We found marginal support for a weak positive relationship between pre-hibernation cortisol and prevalence of WNS disease by histology (LRT: χ^2^ = 4.25, df = 1, P = 0.04).

Model selection results indicate no support for an effect of temperature or humidity on infection response in *P. subflavus* during this captive experiment (Table 2; Fig. 2). In all three model sets, the null model had more support than models with temperature or humidity terms (Table 2). Models that included initial fat mass had equivalent support by AIC to null models for each of the three response metrics (Table 2). Fatter bats at the start of the experiment had slightly lower probabilities of testing positive for *Pd* (LRT: χ^2^ = 3.10, df = 1, P = 0.08) but there was no relationship between initial fat mass and *Pd* loads (LRT: χ^2^ = 0.65, df = 1, P = 0.42) and WNS prevalence (LRT: χ^2^ = 1.69, df = 1, P = 0.19).

There was no evidence of any differences in blood chemistry between bats that tested *Pd*/WNS positive or negative at the end of the experiment (all P > 0.15; Fig. S3). We did not detect differences in total arousals between *Pd*/WNS positive and negative bats (P > 15; Fig. S4).

### Fungal growth on substrates

**Figure 4:**
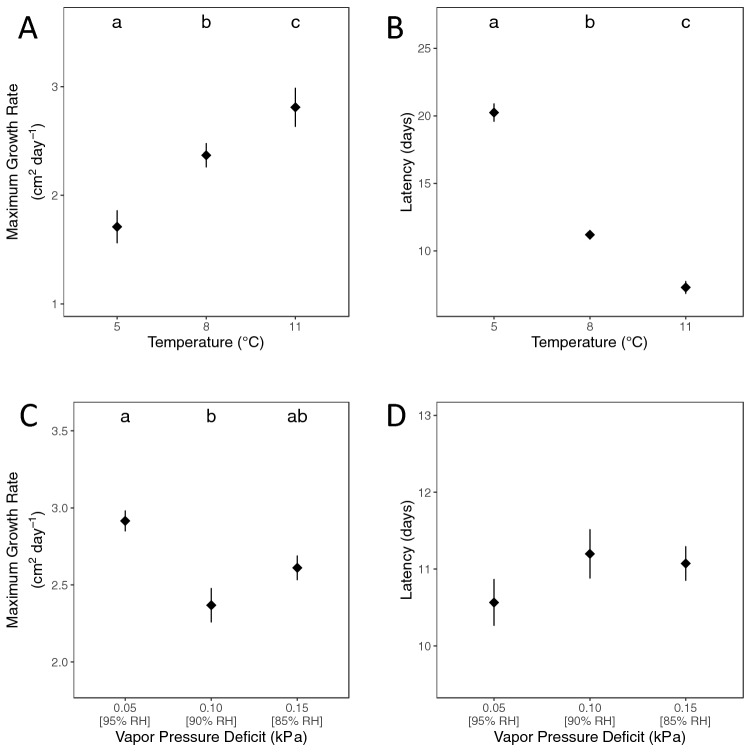
Influence of temperature and humidity on maximum growth rate and latency of growth of *Pseudogymnoascus destructans* (*Pd*) estimated based on the total area of *Pd* fungal growth on media plates. Actual measurements (gray circles) are shown with mean and standard error (diamond with error bars) overlaid. **(A)** Maximum growth rate increased with temperature, and **(B)** latency of growth was significantly shorter at higher temperatures. **(C)** Maximum growth rate was highest at the highest humidity level (vapor pressure deficit at 0.05 kPa) but did not differ significantly from the lowest humidity level (Tukey’s pairwise comparison, P > 0.05), but **(D)** Latency was not significantly different among humidity treatments. Different letters denote that Tukey’s post hoc pair-wise comparisons were significantly different (α = 0.05).

Maximum growth rate on media plates was related to both temperature (F_2,25_ = 19.6, P < 0.0001) and humidity (F_2,25_ = 4.8, P = 0.02), but the effect of temperature was much more pronounced (Fig. 4). Maximum growth rate was positively related to temperature (Fig. 4A; all Tukey’s pairwise comparisons, P < 0.05). The effect of humidity was less pronounced and there was not a consistent effect across humidity. Maximum growth rate was greater at VPD = 0.05 kPa (95% RH) than VPD = 0.10 kPa (90% RH) (Tukey’s pairwise comparison, P = 0.01), with intermediate growth at VPD = 0.15 (85% RH) (Fig. 4C). Latency was not affected by humidity (F_2,25_ = 0.6, P = 0.55; Fig. 4D) but was inversely related to temperature (F_2,25_ = 241.3, P < 0.0001, all Tukey’s pairwise comparisons, P < 0.0001; Fig. 4B).

The results for individual colony growth were similar to the patterns observed for total growth. There was no effect of humidity on maximum growth rate (likelihood ratio = 2.6, df = 2, P = 0.27), but all three temperatures were distinct with growth rate increasing with temperature (LRT: χ^2^ = 48.1, df = 2, P < 0.0001; all Tukey’s pairwise comparisons, p < 0.0001). Similarly, humidity did not affect latency (LRT: χ^2^ = 1.1, df = 2, P = 0.58), but latency decreased with increasing temperature (LRT: χ^2^ = 116.1, df = 2, P < 0.0001; all Tukey’s pairwise comparisons, P < 0.0001).

Although there was no visible fungal growth on the rocks at any timepoint (Fig. S2), fungal load was reliably quantified by qPCR (mean ± SD: 8.03 ± 0.57 (log_10_) ag; range: 5.69 – 9.33 (log_10_) ag). Neither temperature (F_2,110_ = 1.4, p = 0.25) nor humidity (F_2,110_ = 0.47, p = 0.63) affected fungal growth on the natural rock substrate (temperature × day interaction: P = 0.44, humidity × day interaction: p = 0.13). Fungal load increased in the first two weeks (F_3,110_ = 3.6, P = 0.01, Tukey’s pairwise comparison of day 0 to day 14, P = 0.01) and then remained consistent for the remainder of the experiment (all other Tukey’s pairwise comparisons, P > 0.11). After the 8-week experiment concluded, we successfully cultured *Pd* from swabs of the four extreme treatment rocks, confirming the continued viability despite the absence of fungal growth beyond day 14 of the experiment.

## Discussion

We found no evidence that temperature or humidity influenced infection response in *P. subflavus* in this captive hibernation experiment. Why so few bats got infected from experimental inoculation with the fungus is unclear. Nearly two-thirds of the bats showed no signs of *Pd* infection at the end of 83—87 days of captive hibernation (Table 1, Fig. 2), despite being experimentally inoculated with *Pd* using the same protocols as *Pd* infection trials on little brown bats^8,9,15,32,33,37^. The lack of observed infection response from experimental inoculation was an unanticipated outcome and contrasts with extensive evidence that *P. subflavus* in natural hibernation environments experience high *Pd* prevalence, high *Pd* fungal loads, and decline precipitously once *Pd* invades and establishes in hibernacula^11,12,14,20,21,23,24,51,52^.

Individual variation in body condition may be an important indicator of disease susceptibility and overwinter survival for hibernating bats exposed to Pd^53,54^. We used cortisol in fur as a measure of chronic stress and fat mass at the start of the experiment to ask if either of these measures of individual condition were associated with infection response. Generally, we found no support for differences in body condition and infection response in this experiment. However, there was a weak positive relationship between fur cortisol and presence of histological signs of WNS. Future research on how body condition may influence individual susceptibility could inform conservation strategies and may also inform how chronic stressors that may impose physiological costs on bats, such as climate change or reduced prey availability, could be related to disease severity and mortality.

Although the low observed infection response limits interpretation, our experimental design allowed us to gain new insights that temperature is not necessarily the primary condition underlying hibernation energetics^39^ or behavioral selection of microclimate conditions^38^. Decreasing temperature reduces the growth rate of the fungus and decreases energy expenditure in bats^55^, but bats in this captive experiment that could choose their temperature condition generally avoided the coldest condition^38^. Adjustments to hibernaculum microclimates have been proposed as a potential habitat modification aimed to provide beneficial conditions for bat species that have suffered substantial declines^22,24,35^. We caution that bat hibernation is a complex, multidimensional balancing act that warrants different solutions for different individuals and species^27,38,39,56–58^. Further, the way that bats interact with the environment changes throughout hibernation^38,59^.

The results of our experiment concurrently testing the effects of temperature and humidity on growth of *Pd* on artificial and rock substrates showed that *Pd* growth was most strongly related to ambient temperature, with the slowest growth at 5 °C and the fastest growth at 11 °C, near the maximal growth temperature previously reported^26^. The relationship between air moisture and growth of *Pd* had previously only been tested at the temperatures that maximize fungal growth^28^, which tend to be above temperatures where most bats choose to hibernate. Here, we tested the effect of humidity at a temperature (8 °C) more commonly chosen by bats for hibernation. When converted to a physiologically relevant measure of air moisture (VPD), our humidity gradient (VPD: 0.05–0.16) roughly overlaps with the two most humid conditions tested by Marroquin et al^28^ (13.1 °C, 95.5% RH [VPD = 0.068 kPA] and 11.9 °C, 89.5% RH [VPD = 0.146 kPA]). Changes in humidity across this range had little effect on growth of *Pd*. The highest VPD in our experiment was well below the highest VPD (0.446 kPA) tested by Marroquin et al^28^, so we did not detect a decrease in growth in dry air as reported by that study.

The results from our natural substrate experiment are consistent with a growing body of literature that indicates that *Pd* can persist for long periods in the absence of bat hosts^60–62^. Although we observed slight fungal growth over the first sampling period (14 d), there was no change in fungal load over the remainder of the experiment. The growth of *Pd* on natural substrates depends on the interaction of the availability of nutrients and competition with other microbes. For example, *Pd* thrives when grown on sterile guano but growth is prevented by competition from other microbes on fresh guano^63^. We autoclaved the rocks in our experiment to limit competition from other microbes, but a lack of nutrients may have prevented additional growth. Substrates like cave soils or sediments likely provide more nutrients. Indeed, *Pd* has been isolated from cave soils^60^, although growth and persistence in soils can be greatly inhibited by competition from other soil fungi or other microbes^63^.

We explore a few post hoc hypotheses for why infection response was so unexpectedly low. Winter colonies of *P. subflavus* surveyed in culverts in Mississippi have not yet shown typical patterns of the progression of increased *Pd* prevalence and loads and the associated declines in winter colony counts, as has been documented extensively across the range of *P. subflavus* where WNS now occurs^11,21^. The mechanisms of why bats in Mississippi have not yet succumbed to *Pd* invasion and WNS mortality remain unexplored. Given what we know about how *Pd* infection disrupts hibernation physiology and energy balance^64,65^, there is a working assumption that regions with shorter, milder winters could serve as regional refugia from severe impacts^4,66^. However, the idea that hibernating bats may experience less severe impacts in areas with milder winter conditions assumes a behaviorally mediated response from bats being able to spend fewer days in torpor, sustain sufficient euthermic activity to mount an effective immune response, or potentially replenish fat stores with intermittent feeding activity during periodic warm spells^67^. The majority of bats (70/98) in our captive hibernation experiment were placed in controlled microclimate conditions for 83–87 days, which did not permit a behaviorally mediated response. Yet, two-thirds of the *P. subflavus* in this experiment failed to even have detectable levels of *Pd* on their skin tissues at the end of the experiment. Further research would be beneficial to understand what mechanisms could explain why *P. subflavus* in some habitats, such as culverts in Mississippi, have not yet experienced typical WNS progression.

**Figure 5:**
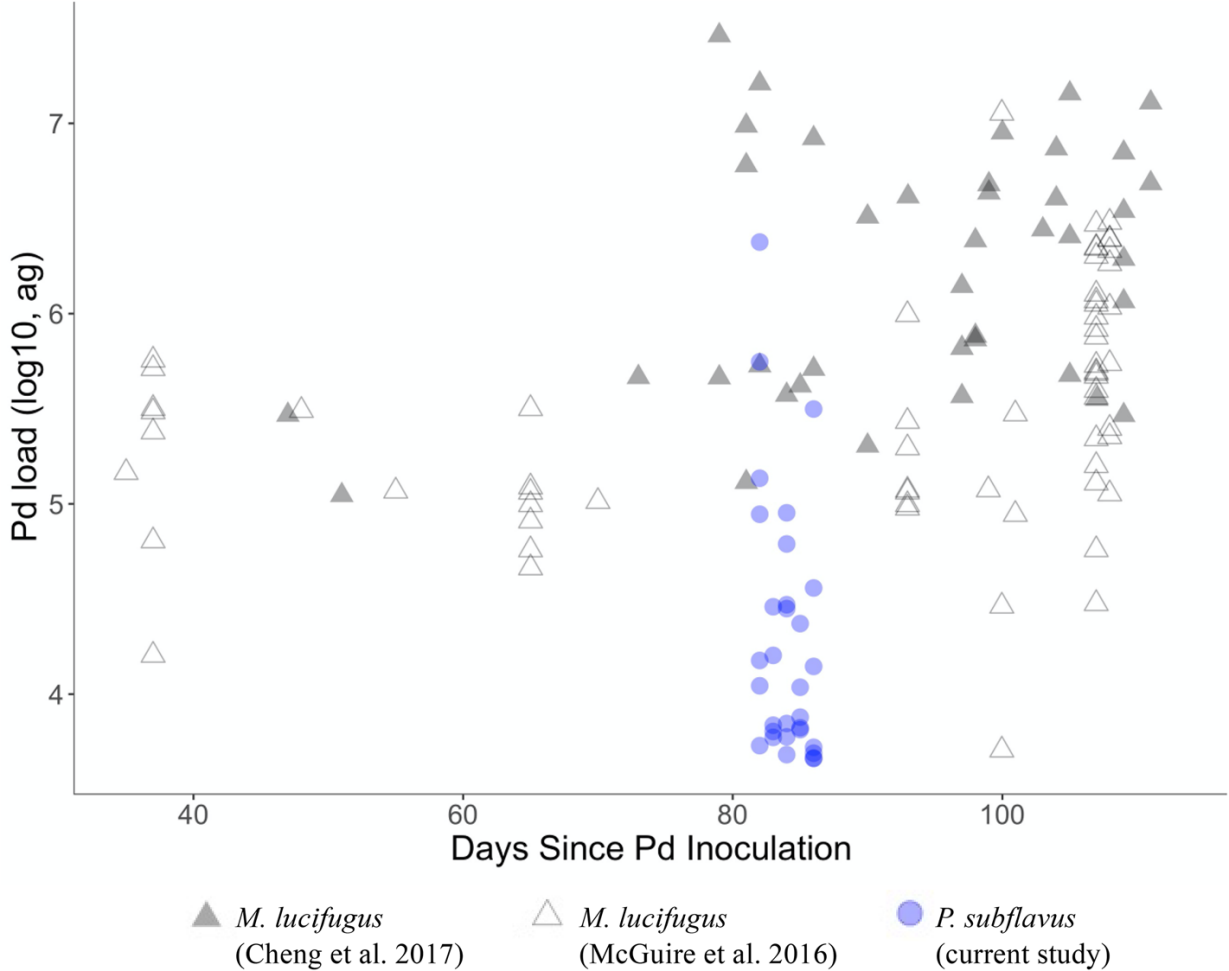
Comparison of fungal loads of *Pseudogymnoascus destructans* (*Pd*) measured on little brown bats (*Myotis lucifugus*) and tri-colored bats (*Perimyotis subflavus*) in experimental infection trials using the same experimental inoculation protocols.

We considered whether our experiment did not run long enough for *Pd* infection and WNS disease to manifest sufficiently. We ended the experiment at 83–87 days based on our best estimate of the duration of bat hibernation in Mississippi, where we collected the animals. We terminated the experiment prior to bats becoming moribund to ensure we were able to collect sufficient data on physiological and disease condition and to comply with IACUC protocols. In captive *M. lucifugus*, death occurs 88–114 days after experimental infection^9^. To compare our results to other similar infection trials with *M. lucifugus*, we plotted *Pd* loads sampled on *M. lucifugus* by days since inoculation from two experimental infection trials conducted with the same inoculation protocols^15,37^ compared to the results of this study (Fig. 5). In general, *M. lucifugus* had higher *Pd* loads, even when sampled as early as 40 days since inoculation. These results indicate that 83–87 days would have been sufficient for *Pd* infection to take hold and fungal loads to increase in a captive setting.

Lastly, our experimental inoculation may have failed for some unidentified reason. While this is plausible, some bats did show infection, indicating this is unlikely. We followed the same general protocols as other experimental infection trials and the inoculation on bats was conducted by some of the same individuals in two previous studies (T. Cheng and L. McGuire). We used a strain of *Pd* cultured from a *M. lucifugus* to guard against the possibility that a laboratorystored strain had lost virulence. It seems unlikely to us that *Pd* cultured from *M. lucifugus* would lack virulence typical of *Pd* infection in the wild, especially given evidence of remarkably low genetic variability in *Pd* from wild-collected samples^68^. The results from our separate experiment testing fungal growth on media plates (Fig. 3) and rock substrates indicated that *Pd* was viable and capable of growth on substrates in the same conditions.

Other studies that experimentally inoculated bats with *Pd* in captive hibernation settings have also experienced challenges in producing results that are consistent with patterns observed in wild populations^8,32^. For example, Johnson et al^32^ used four levels of Pd inoculum and measured survival and *Pd* loads on *M. lucifugus* after hibernating at two different temperatures (4 °C and 10 °C) for 148 days. The results from this experiment were difficult to explain mechanistically given that mortality in the study did not correspond with patterns of *Pd* detection on bats across treatment groups at the end of the experiment.

WNS remains a severe and continuing threat to hibernating bats in North America. Concerted effort has been made over the past 15 years to understand the disease ecology and population impacts and much research attention continues to focus on potential treatments or actions that could improve survival of bats susceptible to the disease. Here, we aimed to augment field studies by providing experimental evidence of the effects of microclimate conditions on the host, pathogen, and environment. Our experiments here and our companion studies of bats hibernating in the same conditions^38,39^ highlight the similar relative influence of temperature and humidity on both host and pathogen when evaluated separately. Temperature has a greater influence than humidity for both host and pathogen and decreasing temperature reduces growth of the fungus and decreases energy expenditure in bats^38,55^. The effects of humidity are less pronounced, but decreasing VPD (i.e., increasing humidity) reduces energy expenditure in bats^39^ while having less effect on the fungus. Behavioral selection of microclimates and the influence of variability in microclimate conditions in natural hibernacula are important to consider in applying these results to inform potential management actions to improve over-winter survival of bats in areas where *Pd* has established and to assess its role in infection as *Pd* spreads into new areas with different environmental conditions.

## Supplementary Information

Supplementary Information.

## Data Availability

The datasets generated by the current study are available from the corresponding author on reasonable request. The histological data and associated metadata are available as a USGS data release via ScienceBase: https://doi.org/10.5066/P9ZM9JIW^69^.
