# Supplementary material for: Experimental inoculation trial to determine the effects of temperature and humidity on White-nose Syndrome in hibernating bats
Source: Sci Rep. 2022 Jan 19;12:971. doi: 10.1038/s41598-022-04965-x (PMC8770465; doi:10.1038/s41598-022-04965-x)
Supplement: Supplementary file 1 — Supplementary Information. [file 41598_2022_4965_MOESM1_ESM.docx]

# SUPPLEMENTAL INFORMATION

**Experimental inoculation trial to determine the effects of temperature and humidity on White-nose Syndrome in hibernating bats**

Winifred F. Frick, Emily Johnson , Tina L. Cheng, Julia S. Lankton, Robin Warne, Jason Dallas^,^ Katy L. Parise, Jeffrey T. Foster, Justin G. Boyles, Liam P. McGuire

# Supplemental Methods

*Cortisol analysis from bat fur*

Bat fur samples weighing between 5 and10 mg were finely minced with scissors and then cortisol was extracted with methanol by mixing with 1.5 ml of methanol in Eppendorf tubes and sonicated in a water bath for 30 minutes at room temperature^1^. The samples were then incubated at 50°C overnight with agitation, after which they were centrifuged at 1500 rpm, to compact the fur. The extracted cortisol-methanol was poured into clean tubes for evaporation under nitrogen in a ReactiVap at 50 °C. The dried cortisol residue was then reconstituted in 200 μl of warmed EIA buffer with 10% ethanol and stored at -20 °C until later EIA analysis. We did not pre-wash the fur because tests have demonstrated pre-washing has no effect on cortisol concentrations^2,3^.

Cortisol concentration (ng/g fur) was measured in the extracted samples using enzyme- immunoassay (EIA) corticosterone kits (Arbor Assays Inc., Ann Arbor, Michigan). These kits were validated using pooled samples for serial dilutions. Samples at a dilution of 1:2 provided an optimal detection range where the serial dilution was parallel to a standard curve (Analysis of Covariance: ANCOVA slope comparison; F1,10 = 0.71, P = 0.43). The coefficient of variation across six assays was 11%, and the minimum percent recovery was 82%.

# Supplemental Figures


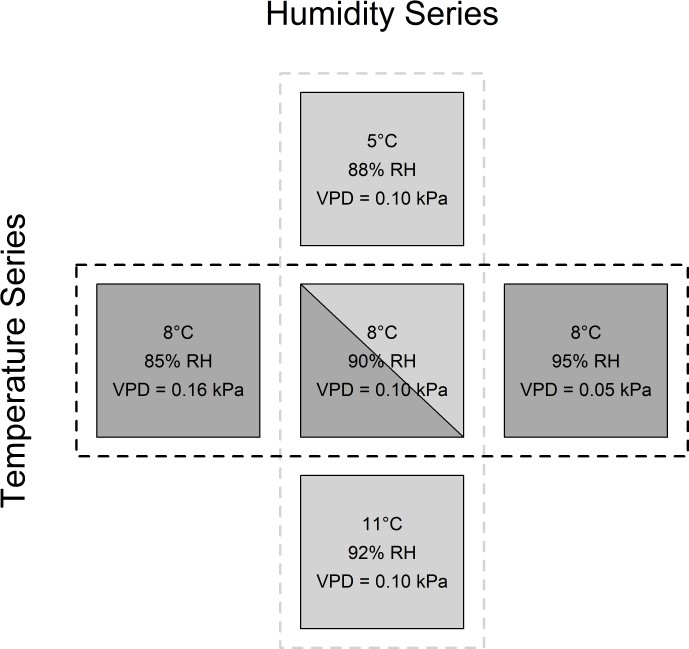


**Figure S1.** Experimental design of the fungal growth on substrates. We present relative humidity for convenience, but experimental design and analysis were based on vapor pressure deficit (VPD). Each square represents an environmental chamber with microclimate conditions listed on each square. Our experiment included a temperature series (light grey) with chambers at 5, 8, or 11°C, all at VPD = 0.10 kPa, and a humidity series (dark grey) with chambers at VPD = 0.16, 0.10, or

0.05 kPa, all at 8°C.


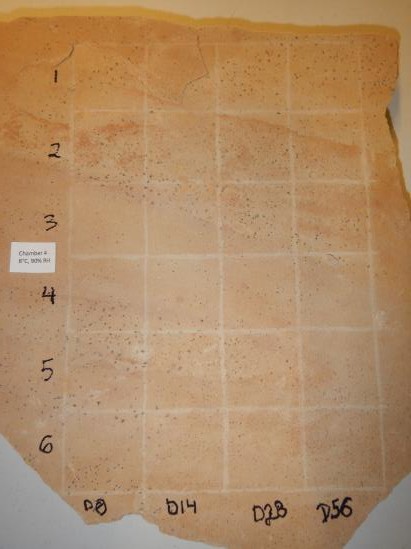


**Figure S2.** To test fungal growth on natural stone, we etched a grid onto stone, defining 6 replicates which were swabbed on days 0, 14,

28, and 56.

# Supplemental Results

**Table S1.** Comparison of blood chemistry and arousals of tri-colored bats (*Perimyotis subflavus*) that tested positive or negative for *Pseudogymnoascus destructans* (*Pd*) or White-nose Syndrome (WNS) at the end of the experiment. For each metric, we show mean values with ± standard deviation (sample size) for bats that were *Pd* or WNS-positive or negative, and the associated P-values from independently run t-tests.

| **Metric** | **Measurement** | **Pd/WNS-**  **positive** | **Pd/WNS-**  **negative** | **P-**  **value** |
| --- | --- | --- | --- | --- |
| blood chemistry | na | 151±6 (28) | 153±8 (40) | 0.27 |
|  | tco2 | 21±4 (28) | 20±3 (40) | 0.16 |
|  | glu | 135±54 (28) | 144±40 (40) | 0.48 |
|  | hct | 50±6 (28) | 51±4 (40) | 0.65 |
|  | ph | 7±0 (28) | 7±0 (40) | 0.21 |
|  | pco2 | 59±8 (28) | 53±9 (40) | 0.014 |
|  | hco3 | 20±3 (28) | 19±3 (40) | 0.24 |
|  | be | -9±4 (28) | -10±4 (40) | 0.4 |
|  | angap | 7±5 (28) | 6±6 (40) | 0.7 |
|  | hgb | 17±2 (28) | 17±1 (40) | 0.61 |
|  | cl_modified | 134±10 (28) | 137±11 (40) | 0.3 |
|  | bun_modified | 90±35 (28) | 96±32 (40) | 0.48 |
|  | k_modified | 7±1 (28) | 7±1 (40) | 0.87 |
| arousals | total arousals | 5±2 (35) | 6±2 (58) | 0.17 |


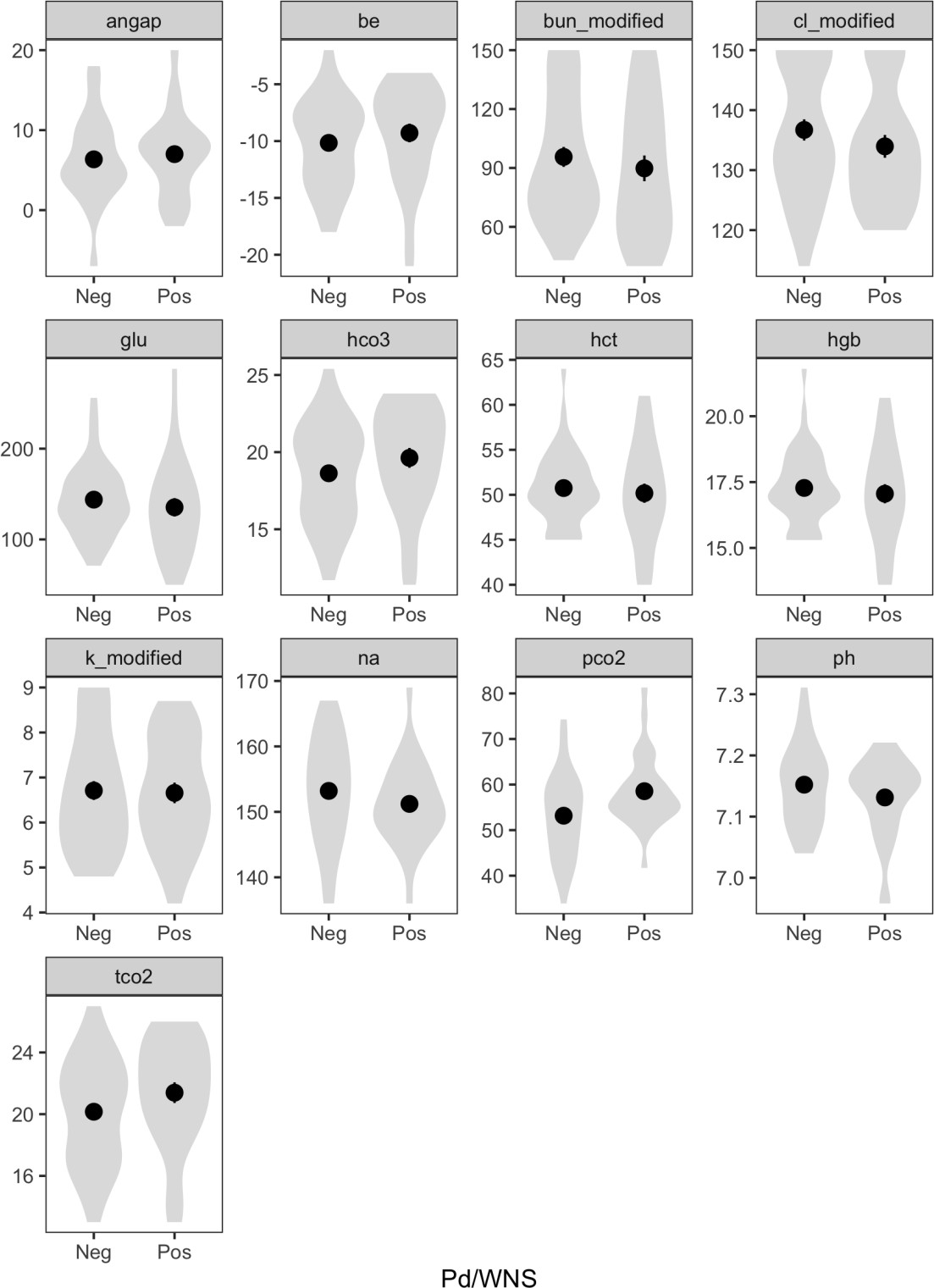


**Figure S3.** Comparison of 13 blood chemistry metrics sampled from tri-colored bats (*Perimyotis subflavus*) that tested *Pd*/WNS positive or negative at the end of the experiment. Data visualized as violin plots with gray polygons showing the kernel probability density of the data at different values, and means and standard errors are plotted as points and vertical lines.


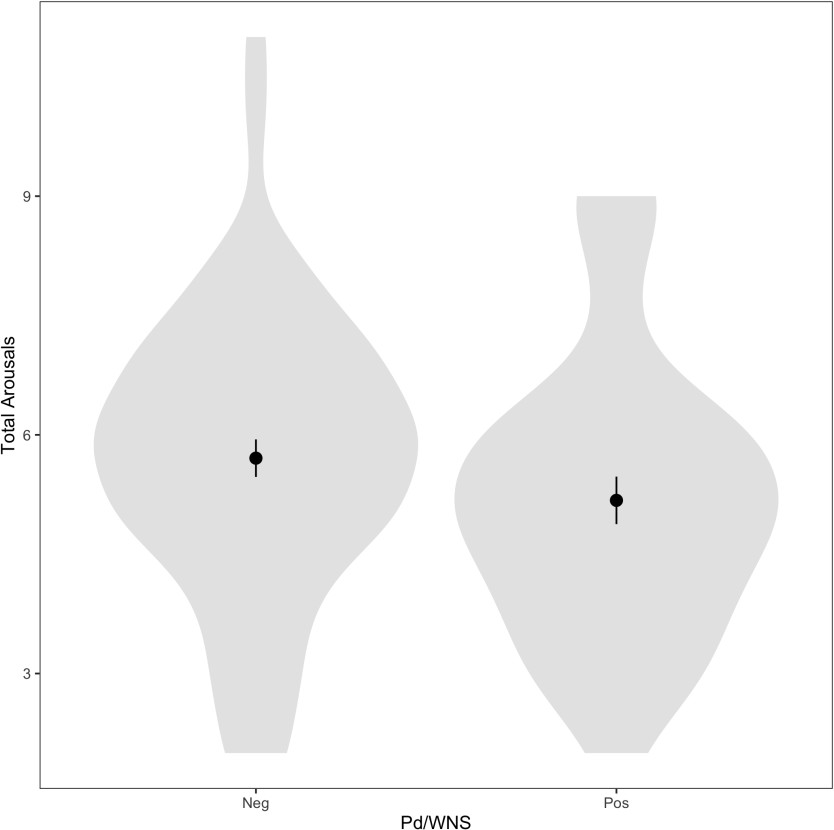


**Figure S4.** Comparison of total arousals of tri-colored bats (*Perimyotis subflavus*) over the duration of the experiment for bats that were *Pd*/WNS positive or negative at the end of the experiment. Data visualized as violin plots with gray polygons showing the kernel probability density of the data at different values, and means and standard errors are plotted as points and vertical lines.

The use of trade, firm, or product names is for descriptive purposes only and does not imply endorsement by the U.S. Government.

# References

1. Warne, R. W., Proudfoot, G. A. & Crespi, E. J. Biomarkers of animal health: integrating nutritional ecology, endocrine ecophysiology, ecoimmunology, and geospatial ecology. **5**, 1–10 (2015).
2. Slominski, R., Rovnaghi, C. R. & Anand, K. J. S. Methodological considerations for hair cortisol measurements in children. **37**, 812–820 (2015).
3. Albar, W. F., Russell, E. W., Koren, G., Rieder, M. J. & Umm, S. H. V. Human hair cortisol analysis: comparison of the internationally-reported ELISA methods. E312–E316 (2013).
